# Comparative GO: A Web Application for Comparative Gene Ontology and Gene Ontology-Based Gene Selection in Bacteria

**DOI:** 10.1371/journal.pone.0058759

**Published:** 2013-03-11

**Authors:** Mario Fruzangohar, Esmaeil Ebrahimie, Abiodun D. Ogunniyi, Layla K. Mahdi, James C. Paton, David L. Adelson

**Affiliations:** 1 Centre for Bioinformatics and Computational Genetics, School of Molecular and Biomedical Science, The University of Adelaide, Adelaide, South Australia, Australia; 2 Research Centre for Infectious Diseases, School of Molecular and Biomedical Science, The University of Adelaide, Adelaide, South Australia, Australia; UC Davis School of Medicine, United States of America

## Abstract

The primary means of classifying new functions for genes and proteins relies on Gene Ontology (GO), which defines genes/proteins using a controlled vocabulary in terms of their Molecular Function, Biological Process and Cellular Component. The challenge is to present this information to researchers to compare and discover patterns in multiple datasets using visually comprehensible and user-friendly statistical reports. Importantly, while there are many GO resources available for eukaryotes, there are none suitable for simultaneous, graphical and statistical comparison between multiple datasets. In addition, none of them supports comprehensive resources for bacteria. By using *Streptococcus pneumoniae* as a model, we identified and collected GO resources including genes, proteins, taxonomy and GO relationships from NCBI, UniProt and GO organisations. Then, we designed database tables in PostgreSQL database server and developed a Java application to extract data from source files and loaded into database automatically. We developed a PHP web application based on Model-View-Control architecture, used a specific data structure as well as current and novel algorithms to estimate GO graphs parameters. We designed different navigation and visualization methods on the graphs and integrated these into graphical reports. This tool is particularly significant when comparing GO groups between multiple samples (including those of pathogenic bacteria) from different sources simultaneously. Comparing GO protein distribution among up- or down-regulated genes from different samples can improve understanding of biological pathways, and mechanism(s) of infection. It can also aid in the discovery of genes associated with specific function(s) for investigation as a novel vaccine or therapeutic targets.

**Availability:**

http://turing.ersa.edu.au/BacteriaGO.

## Introduction

Thousands of papers describing new functions for genes and proteins are published every year, and integrating these results into a useful knowledgebase is an ongoing challenge. The primary means of classifying these results relies on Gene Ontology (GO), which was initially invented to unify the representation of gene and gene product attributes across all eukaryotes using a set of structured, controlled vocabularies [1]–[3]. The main goal of GO is to develop ontologies to support biologically meaningful annotation of genes and their products in terms of their Molecular Function (MF), Biological Process (BP) and Cellular Component (CC) [1]–[3]. A list of GO terms can be easily used to build a graph describing the relationship between said terms. Alternatively, text-mining tools using entity recognition methods, combined with manual curation, can be used to extract GO terms associated with a list of genes or proteins. Moreover, the concept of GO network interaction in addition to gene network interaction has recently been developed in model eukaryotes including human, mouse, and Arabidopsis using advanced web applications such as COXPRESdb (http://coxpresdb.jp/) and ATTED-II (http://atted.jp/). This new concept has provided more comprehensive analytical approach in systems biology.

While quality-based gene selection strategies such as GO are established in eukaryotes [4], [5], the common approach of gene selection in bacteria is based on level of gene expression (quantity-based gene selection). However, the quantity of expression can not be assumed as a sole index of gene significance as some genes with common lower amount of gene expression (such as transcription factors) play a prominent role in bacterial systems biology. Therefore, the use of quality-based metrics such as promoter architecture, GO classification, and network analysis in conjunction with quantity-based gene selection criteria provides a more robust approach for elucidating key bacterial genes and unraveling bacterial systems biology. The challenge is to present this information to researchers to compare and discover patterns in multiple datasets using user-friendly visual statistical reports. Furthermore, reliable non-parametric statistical tests need to be integrated into GO web applications in order to compare GO distribution of multiple samples.

To fill these needs, we have designed a web server to compare GO protein distributions from gene expression data, using *Streptococcus pneumoniae* as a model. This organism serves as a paradigm for bacterial pathogens that colonize mucosal surfaces (such as the nose and throat) without causing symptoms, prior to invasion of deeper host tissues, such as the lungs, blood and brain [6], [7]. For the first time, we have implemented non-parametric (Kolmogorov–Smirnov [K–S] and Wilcoxon Rank Sum) tests [8]–[10] to compare GO distribution of multiple samples and Goodness-of-Fit (Chi-square and K–S) tests to compare one sample against its expected reference genome distribution. This application is particularly significant when comparing GO distribution between samples from different sources, such as gene expression patterns *in vitro* vs *in vivo*, or between one anatomic niche and another, for example, gene expression patterns between bacteria harvested from an initial site of infection (such as the nose) and expression patterns during translocation into deeper host tissues, such as lungs, blood, or brain [11], [12]. Comparing GO protein distribution among list of up- or down-regulated bacterial genes from different samples can help to understand biological pathways, and mechanism(s) of pathogenesis. It can also help to detect a gene that has been associated with a specific function, and investigate this as a novel vaccine or therapeutic target.

To our knowledge, while there are many GO resources available on the web [13]–[17], none are suitable for comparison of multiple datasets and gene selection and none contain bacterial data. Our web server is able to rapidly compare large lists of genes/proteins with respect to their GO protein distributions and is regularly updated with the latest gene/protein and GO data.

## Materials and Methods

### Web Application Architectural Design

**Figure 1:**
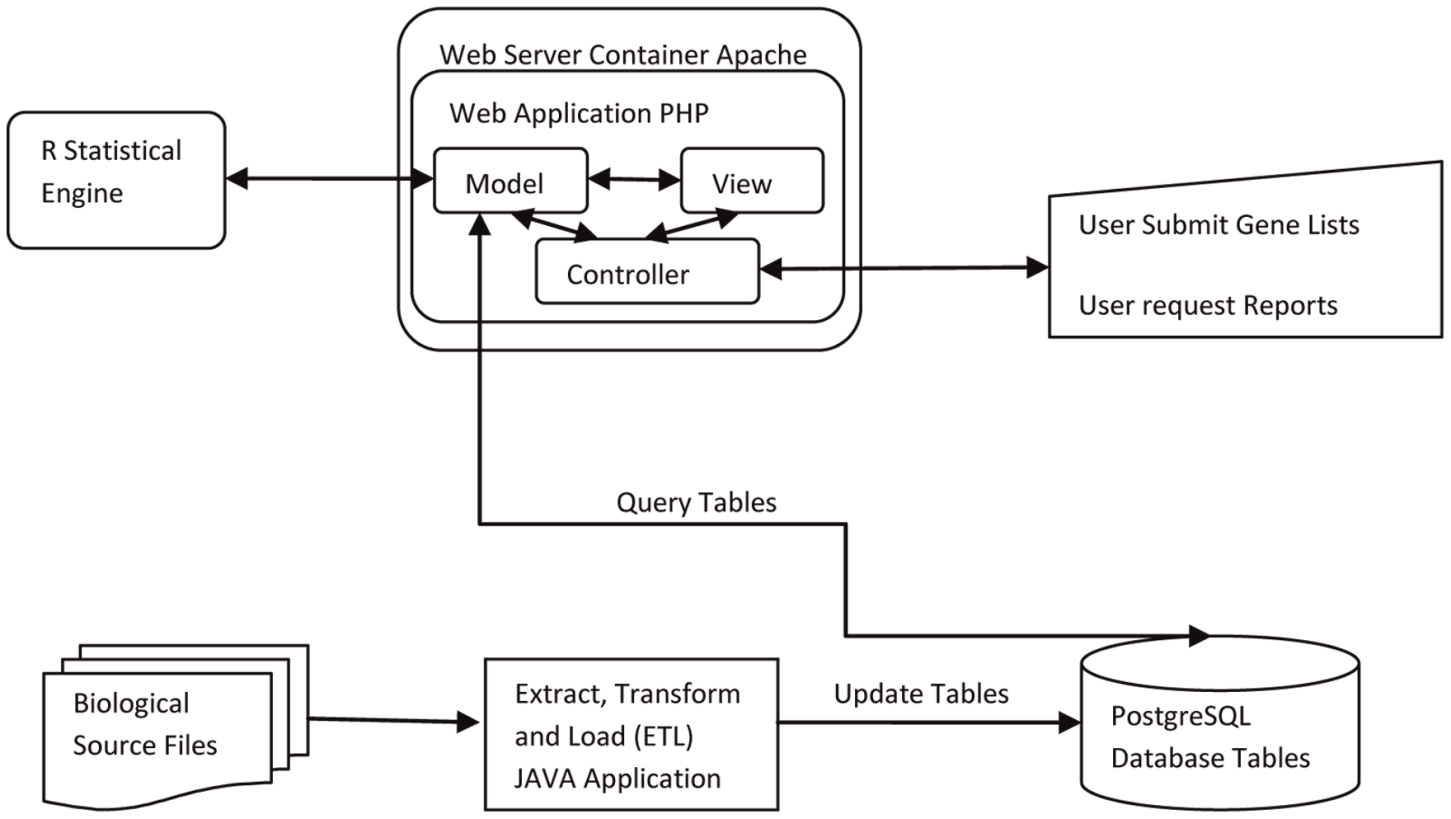
Schematic component diagram of the application. PostgreSQL database is in the centre of system. Lower part of diagram illustrates updating database, and upper part shows how web application uses database.

In order to obtain a user-friendly and statistically meaningful web application to compare and discover patterns in multiple gene lists, we built a web application based on advanced technological standards. The overall schematic component diagram of the application is shown in Figure 1. In the lower part of Figure 1, there is a process of updating database table. This process ensures that latest protein, gene and GO data exists in the main database system. The main part of system is a web application that is hosted under apache web server. The web application consists of 3 major parts: Model, View and Controller (MVC). Model part contains all database and table query operations, and business logic. It is also responsible to interact with R statistical engine. View part contains all visual components and client side logic including Ajax, JavaScript and HTML. View, with the help of Model, can generate required report to be sent to Controller that interacts with user. Controller part contains all the logic regarding handling user HTTP requests and sending back response to user and also it orchestrates Model and View operations. In other words, it makes instances of objects from View and Model and calls their methods in turn, to send output to user.

### Data Collection and Sources

We collected and classified all data needed for the system as:

*Gene Ids, gene class name (Primary, Synonym, ordered-locus, ORF) and protein names beside protein accession numbers.* Collected from uniprot.org ftp server, we processed manually curated file (uniprot_sprot) and automatically generated file (uniprot_trembl).

*Gene ontology Ids and descriptions beside GO relationships (is_a, has_part, part_of, regulates, occurs_in, positively_regulates, negatively_regulates).* Collected from geneontology.org ftp server [18].

*Protein-GO relationships.* Collected from uniprot.org ftp server [19].

*Taxonomy Ids and descriptions.* Collected from ncbi.nlm.nih.gov ftp server.

### Data Storage and Update

**Figure 2:**
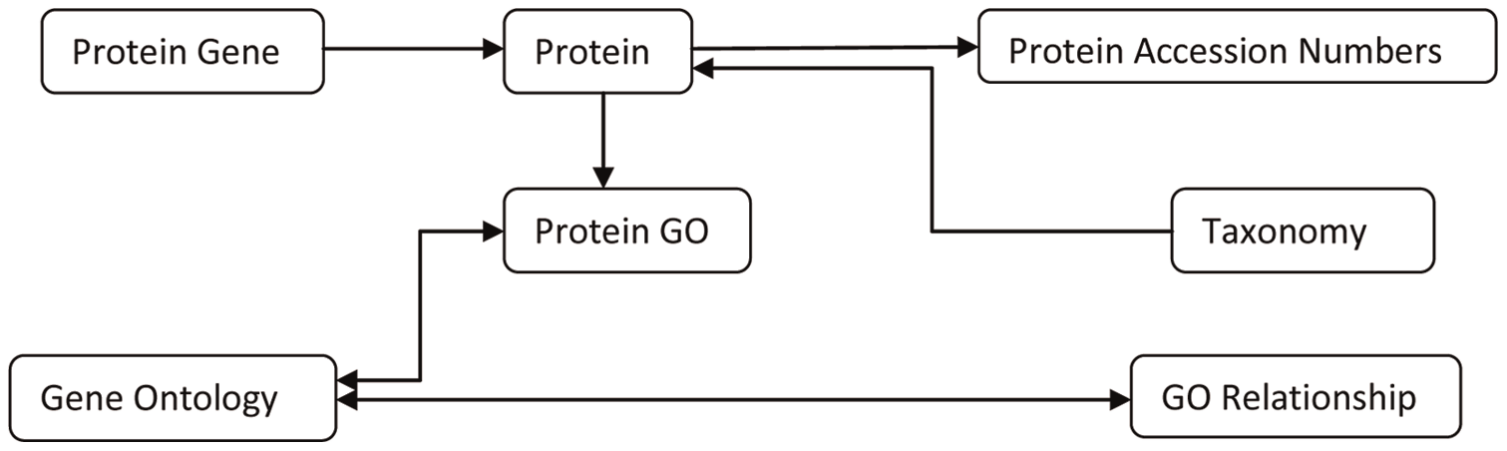
Entity Relationship Diagram. Each entity represents a database table in the system, arrows between entities represent type and multiplicity of relationship between them.

We stored all the data collected, in a PostGreSQL database, in 6 main tables in normalized form, depicted as an ER (Entity-Relationship) diagram, in Figure 2. For better performance, we have created multiple indexes on all searchable fields. We have used joint table queries as much as possible to improve database performance, and cut down number of queries. We developed application in Java to download flat files from mentioned sources and update tables every 2 weeks automatically.

### Data Structures and Processing Logic

**Figure 3:**
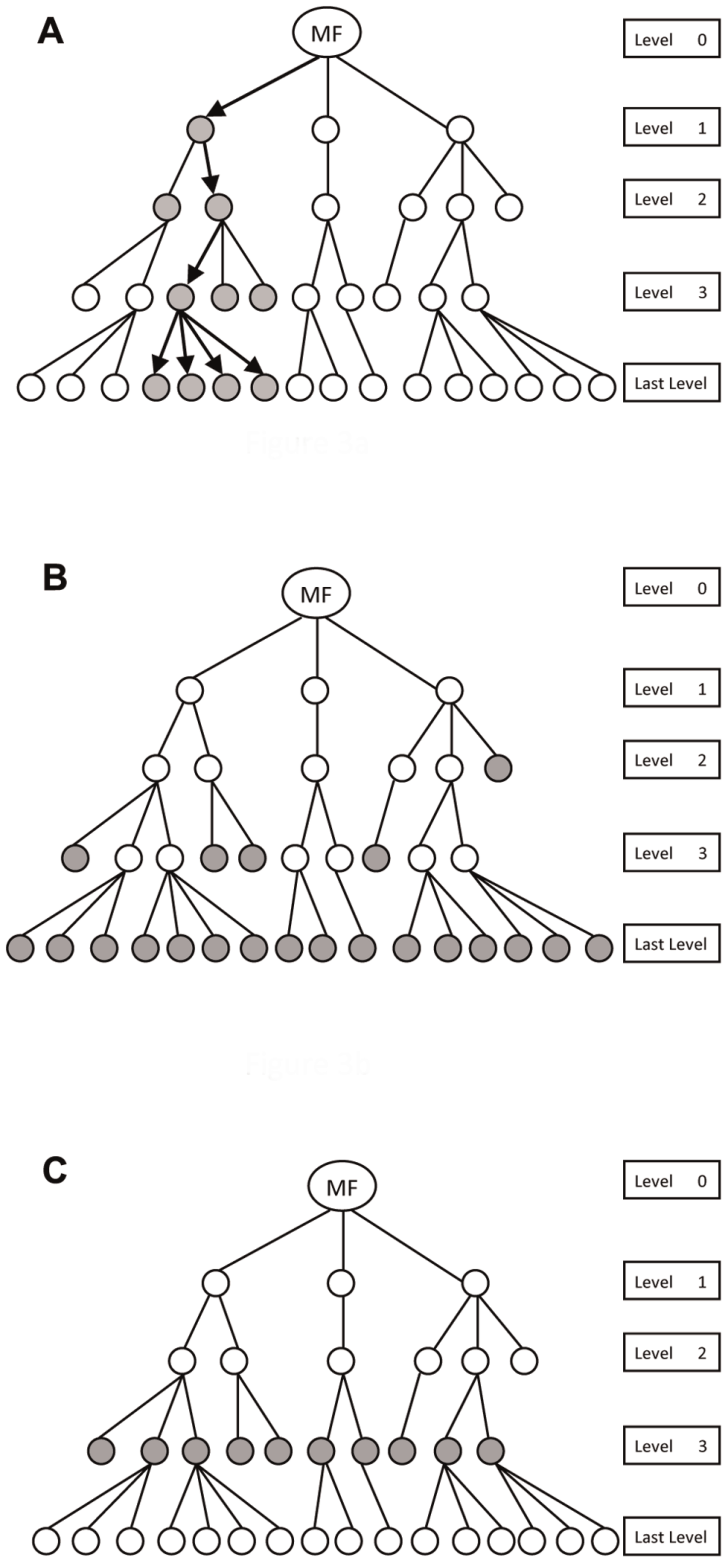
GO Graphs, Navigation and Visualization. A graph structure of molecular function (MF) is built in the memory for one sample. Navigation is done from root (level 0) to the leaves (last level) and vice versa. Visualization can be done in three ways: (A). From each node in a specific level, children of that node are visualized. (B). Leaves of the graph or the most details GO terms are visualized (C). At each level of a GO graph, all the nodes at that level are visualized.

In order to prepare useful and user friendly reports, we developed logic and data structures in PHP and integrated into Model part of the web application. The major data structure was directed acyclic graph (or tree, if each node does not have more than one parent node) made from gene ontology and GO relationships. The graph was implemented using linked lists. This data structure is rather large, so we imposed strict PHP memory management to minimize the memory used by the process. Nodes of the graph contain gene ontology and related gene, Protein Ids and other useful information. Root of the graph is one of 3 name spaces MF, BP or CC. Navigation across this graph can be done in multiple ways to produce different reports, which results in proper biological inference. This organization allows for novel visualization of GO graph. In Figure 3, we illustrate how the user can observe nodes of graph and how to navigate through the graph. First, we assign a level to each node of graph starting from root node (level 0), and nodes next to it level 1 and nodes by 2 edges distant level 2, and so forth. The leaves of the graph (nodes that have no children) represent most detail GOs. According to the leveling method, leaves of the graph can be located in multiple levels, not essentially in deepest level (Figure 3B).

In Figure 3A, graph is navigated from root to leaves and vice versa. If current node is in level i, the children nodes of current node (which are located in level i+1), are visualized. Arrows and grey nodes explain logic of navigation. We will explain how informative this navigation method could be in comparing multiple GO graphs in the form of pie charts. In Figure 3B, leaves of the graph are shown as grey nodes. These leaves bear the most detailed GO information. This navigation is supported in all of the visualization reports. In Figure 3C, graph is navigated from root to leaves and vice versa, and at each level, all the nodes in that level is visualized. We will demonstrate how helpful this navigation could be in gene selection mechanism. In this application, all the hypothesis testing and statistical processing is performed using R statistical package. R is externally called by PHP web application. We developed a parameterized R script that can be externally executed and passed by parameters to do statistical analysis.

### Genome Wide Comparison and Reference Genome Size Estimation

In order to perform comparison between a gene list and its genome, we used hyper-geometric distribution. This comparison reveals whether a particular GO in a gene list is over-represented or under-represented. For a better user experience, we estimated whole reference genome automatically from database using a novel method, so unlike other web applications user does not need to submit reference genome manually. Other GO web applications prepare 2 by 2 contingency table for each GO group at a time and perform Fisher exact test and report significant GOs based on *P*-value of the test. Instead, we have presented all the common GO groups between sample and its reference genome in a novel bar chart as observed protein numbers next to the expected protein number. Eventually, K–S test is used to compare all GO groups at once between sample and reference genome. Expected protein number of each GO group in sample *i*, represented by E(GO*_i_*), is mean of hyper-geometric distribution [20]:
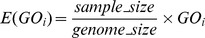



To estimate genome size of a taxonomy, we developed a novel method. We first counted number of gene Ids and classified it based on class name (Primary, Synonym, ordered-locus, ORF). We picked the class name with highest number of counts. This number very likely represents actual number of genes in genome. For example we performed this method in *S. pneumoniae*. Estimated genome size 2115 genes pertaining to Ordered-Locus name class, where this number is very close to actual numbers.

### Normalization of Protein Numbers of GOs in Multiple Samples

When samples have different number of genes, in order to compare protein numbers of one GO in all samples, we need to adjust protein number based on sample size. We used a simple method. In this method we estimate a coefficient for each sample. Instead of actual protein number we consider product of coefficient of the sample by actual protein number. To estimate coefficients, we order samples based on their size as S_1_……S_n_ with the lengths of *l_1_……l_n_.* We assign 1 to coefficient of biggest sample (S_1_), then for the rest of samples we have:
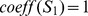


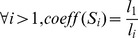



### Data Presentation and Visualization

This part of the application implements ‘View’ part of MVC framework. We have used open source PHP components to produce graphs and charts mainly in Jpg image format. According to our experience, using image-rendered graphs is not only faster than Flash and other plug-ins, but also demands much less memory and CPU on the web browser. Besides, all of plug-ins impose dependency, whereas Jpg images are supported in all browsers and there is no need for manual installation of a plug-in. For a better user experience, we used Ajax as much as possible. Specifically, wherever comparisons are performed among multiple gene lists, data related to each gene list is visualized separately in its own HTML division (div) element, where each division is built and updated separately by one Ajax script. At waiting times, when the application is doing a long running job, an Ajax progress bar component is used.

## Results

Unlike other GO tools, our application is specifically designed to generate novel reports to compare multiple gene lists. These reports enable researchers have better understanding of biological pathways, and mechanism(s) of pathogenesis. In addition, it can also help to detect a gene that has been associated with a specific function, and investigate this as a novel vaccine or therapeutic target. To demonstrate the usefulness of this application, we have compared RNA expression of *S. pneumoniae* harvested from the nose lungs, blood, and brain of infected mice. Example data is available on the web application home page to reproduce the reports. We prepared multiple lists of up- and down-regulated pneumococcal genes from various niches and analyzed these lists in the web application using a selection of reports described below.

### (A) Pie Chart Comparing Multiple Samples GO Distribution

**Figure 4:**
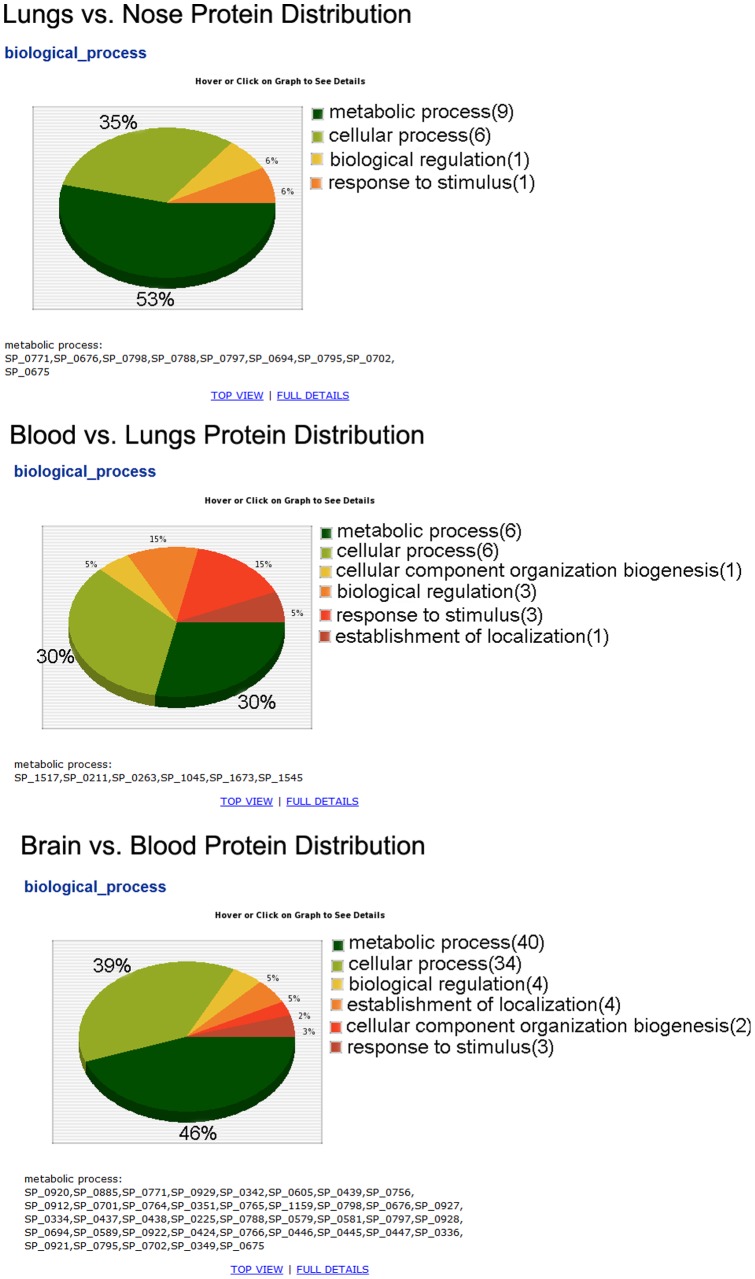
Pie Chart illustrating multiple samples protein distribution. Change of ‘metabolic process’ protein distribution (percentage) can explain level of bacterial activity in each tissue.

One of the better methods to visualize change of a specific GO in multiple gene lists is to present percentage of protein distribution among gene lists in pie chart. In the example data provided (Figure 4), we used this novel method to investigate protein distribution involved in “metabolic process” (equivalent to Figure 3A, level 1) between three gene lists from the three comparisons. The results show that the proteins involved in metabolic process constituted 53%, 30%, and 46% of all proteins in the lungs, blood, and brain, respectively. This suggests that pneumococcal genes involved in metabolic process are under-represented in blood during pathogenesis. This report enables user to navigate and observe GO graphs according to Figure 3A. The report also shows related genes in each GO item of the pie chart.

### (B) Graph comparing sample versus genome GO distribution

**Figure 5:**
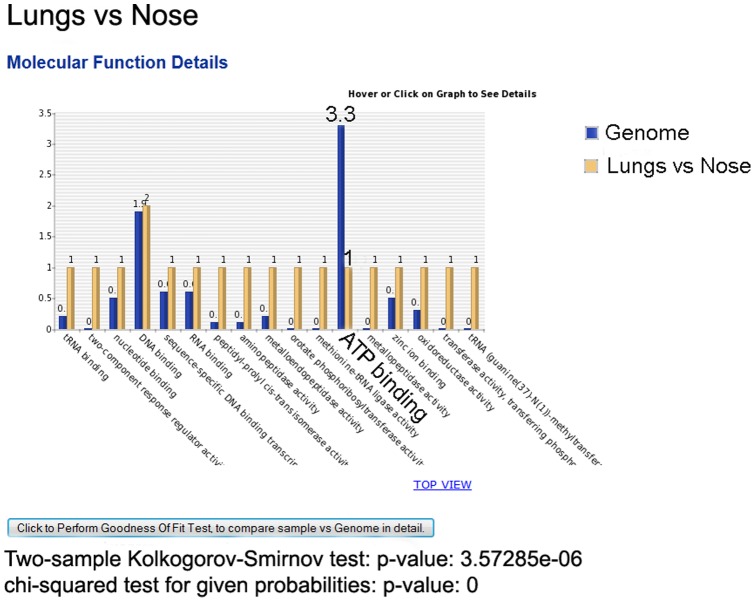
Bar Chart comparing a sample versus its Genome protein distribution. ‘ATP binding’ protein level of sample is substantially less than its expected number based on whole Genome.

As we mentioned in the Materials and Methods under Genome Wide Comparison and Reference Genome Size Estimation section, comparing gene lists with their expected genome-wide protein distribution can give insight into potential biological significance, especially when this comparison is confirmed by statistical hypothesis testing. Figure 5 shows an example of this capability using bar chart. Here, in the lungs vs nose comparison, under “Molecular Function” ATP binding' molecular function is substantially less than its expected genome distribution. Goodness of fit statistical tests (K–S and Chi-square tests) for all GO items at the Molecular Function level, are also reported in the figure. Unlike Pie chart, user can only see number of proteins, instead of percentage of proteins, in each GO.

### (C) Tabular gene ontology visualization for gene selection

**Figure 6:**
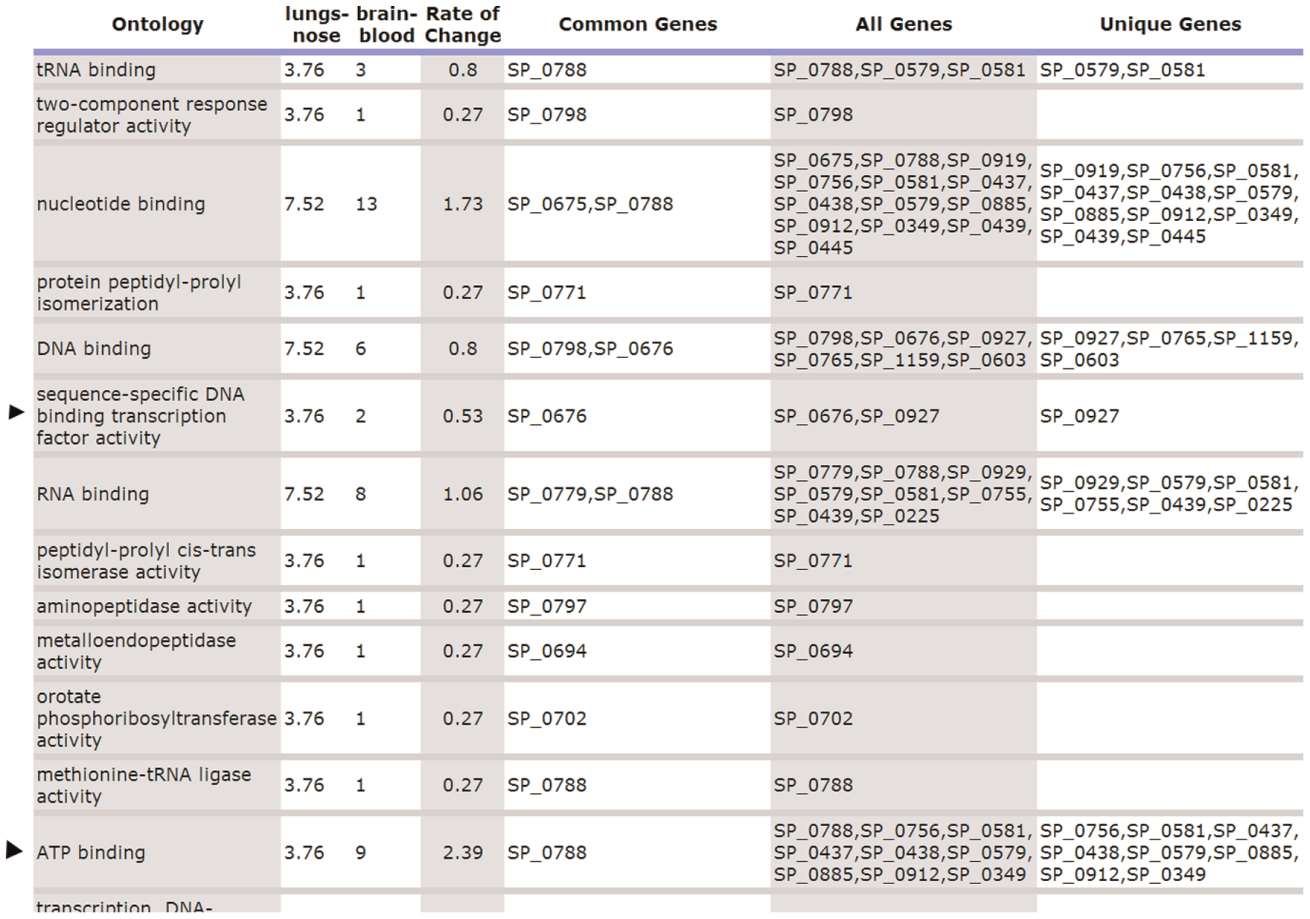
Tabular Report Used in Selecting Important Genes and Important GO. Genes located under ‘common genes’ column are the most important genes in each GO item. On the other hand, GO item with higher ‘Rate of Change’ represents more significant biological trend.

In our first trial of this system, it was confirmed that this novel report is very effective in selecting important genes. Navigation through the GO graph in this report is based on Figure 3C. In other words, unlike Pie chart user can observe protein distribution of all the GO nodes at a given level of the tree. User can also navigate to leaves of the graph to observe the most detail information as shown in Figure 3B. In this report, each GO item at a given level is shown across selected gene lists (extracted from multiple biological samples) in a line. So, one can observe the rate of change in one GO (by time or location of sample). If there is a significant rate of change in one GO, it is selected for further investigation. Along with rate of change, common genes (intersection) and all the genes (union) are also reported. Common genes can be particularly important, because those genes are over-represented in all gene lists for a given GO. One example of this report is depicted in Figure 6, where lungs vs nose and brain vs blood gene lists were compared. Here, we can observe that ‘Sequence-Specific DNA binding transcription factor activity’ (arrowed) has been significantly reduced with 0.53 rate and the gene responsible for this is SP_0676. Another example is ‘ATP binding’ (arrowed) which increased 2.39 times, and the common gene responsible for that is SP_0788.

## Discussion

GO analysis provides a new avenue for deeper understanding of gene expression and function, which can be exploited in the context of quality-based gene selection strategy. To achieve this goal, comparative statistically based comparison of GO groups and enriched database are crucial. Current GO web applications are mostly employed in eukaryotic genomes, and lack of reliable comparative statistical analytical approaches hinder the application of GO concept in bacteria. To fill this need, we have designed a user-friendly web application to compare GO protein distributions from gene expression data, using *S. pneumoniae* as a model.

For the first time, we present a dynamic pie chart that illustrates different GO groups as well as the genes involved in each group. This approach allows the user to have a clear, visual comparative understanding of GO distribution in all levels of GO graphs. This can unravel the underlying differential biological pathways, metabolic activation groups, and regulatory networks. Such comparative GO assignments can significantly increase our knowledge of functional genome arrangement and shift during pathogenesis, and provides an avenue for predicting possible activated functional GOs of future virulent strains.

Other GO web resources are able to compare one sample against another reference sample with respect to one GO group at a time, and report the result of enriched GOs based on *P*-values (using Fisher exact test and Chi-square). Instead, our application is able to compare multiple samples visually and statistically using pie chart and solid non-parametric statistical tests (K–S test and Wilcoxon Rank Sum Test) to compare whole samples against each other considering all GO groups. We also managed to facilitate the process of data entry and submitting gene lists. In addition, the Goodness-of-Fit test compares the distribution of GO groups between any given sample versus the reference genome. This test provides another piece of information for finding over- or under-represented GO groups, relative to the entire genome. Such discovered GO groups offer a route map for prevention and/or treatment of bacterial pathogenesis and virulence through inactivation of the GO group.

In this work, we also present tabular GO visualization for gene selection. This simple approach offers the advantage of finding genes that are common to samples from different sources (for example, genes that are central to a pathogenic process). Such genes would serve as targets for controlling the movement of pathogens from one tissue to another. The tabular data also presents the rate of change in number of genes/proteins between samples, which has significant implication in deciding which functional GO group is more enriched between given samples. For example, functional groups involved in two-component sensor activity, DNA binding, and antioxidant activity, are central to *S. pneumoniae* functional genomics. These groups are excellent targets for monitoring bacterial evolution and pathogenesis and provide valuable clues for predicting the possible activated GOs of emerging virulent strains.

In conclusion, we present a novel, user-friendly web application that compares GO protein distributions across samples from different sources. This tool is particularly useful in understanding biological pathways, mechanism(s) of infection and discovery of genes associated with specific function(s) for investigation as a novel vaccine or therapeutic targets. The application can also be expanded to integrate gene expression levels (quantity-based) with the current quality-based GO approach, which would result in more accurate selection of important genes from diverse biological sources. It also has the potential to include eukaryotic information to study diseases such as human cancer and other biological phenomena.
