# Correction: Comparative GO: A Web Application for Comparative Gene Ontology and Gene Ontology-Based Gene Selection in Bacteria

**DOI:** 10.1371/journal.pone.0125537

**Published:** 2015-04-17

**Authors:** Mario Fruzangohar, Esmaeil Ebrahimie, Abiodun D. Ogunniyi, Layla K. Mahdi, James C. Paton, David L. Adelson

There is an updated URL for the Comparative GO website. The correct, complete URL for Comparative GO is: http://www.comparativego.com/. The Availability subheading within the Abstract should read as follows: http://www.comparativego.com/.
